# Impact of virtual ICU implementation on clinical outcomes across multiple critical care units: A before-and-after study

**DOI:** 10.1371/journal.pdig.0001186

**Published:** 2026-01-16

**Authors:** Annemarie Nguyen, Sprague W. Hazard, Anthony S. Bonavia

**Affiliations:** 1 Penn State College of Medicine, Hershey, Pennsylvania, United States of America; 2 Division of Critical Care Medicine, Department of Anesthesiology and Perioperative Medicine, Penn State Hershey Medical Center, Hershey, Pennsylvania, United States of America; Iran University of Medical Sciences, IRAN, ISLAMIC REPUBLIC OF

## Abstract

Virtual intensive care units (vICUs) provide continuous remote monitoring and support for critically ill patients. Increasing patient complexity and staffing shortages have driven interest in vICUs, but evidence of their impact on clinical outcomes is limited. This study evaluated the effect of vICU implementation across critical care units in a large academic medical center. We conducted a before-and-after study comparing outcomes during the initial vICU implementation period (October 2022–April 2023) and the established program period (October 2023–April 2024), with a 6-month washout interval. Adult patients from a multispecialty surgical intensive care unit (ICU), neurocritical care unit, and ICU step-down unit were included if they had ICU stays longer than 6 h, hospital stays under 30 days, and mechanical ventilation for at least 12 h. The primary outcome was ICU length of stay, with secondary outcomes including hospital stay, ventilation time, vasopressor use, readmissions, and mortality. Among 530 patients (266 implementation, 264 established), ICU length of stay decreased from 232 to 198 h (p=0.011), ventilation time from 110 to 90 h (p=0.044), and vasopressor use for more than 12 h from 55% to 43% (p=0.007). Hospital stay, mortality, and readmission rates were unchanged. Subgroup analysis showed the greatest improvements in the surgical ICU, with reductions in ICU stay, ventilation time, and vasopressor use. These improvements may reflect earlier recognition of deterioration, better care coordination, and timely withdrawal of intensive therapies. Variation across units highlights the need to tailor vICU integration strategies to specific clinical workflows. These findings suggest that vICU implementation is feasible and may enhance critical care efficiency, though further multi-center studies are needed to determine generalizability and to assess patient-centered and economic outcomes.

## Author summary

We investigated whether a virtual intensive care program could improve the care of critically ill patients in a large hospital. This program allows experienced clinicians to monitor patients remotely and to provide guidance to the primary clinical care team. We compared results from when the program first started to when it was fully in place. In the later period, patients spent less time in the intensive care unit, needed breathing support for fewer days, and needed blood-pressure–supporting medicines for a shorter time. These benefits were especially evident in patients recovering from surgery. Importantly, overall hospital stay, death rates, and readmissions remained similar, suggesting the improvements were specific to critical care management. Our findings indicate that virtual intensive care may help identify patient issues earlier, improve coordination of care, and reduce prolonged intensive treatments. This work lays the foundation for future studies examining how virtual care can enhance patient recovery and hospital efficiency.

## Introduction

Over the past decade, artificial intelligence (AI) has rapidly integrated into healthcare, with promises of improved outcomes, efficiency, and cost-effectiveness [1]. In critical care, particularly intensive care units (ICUs), AI reflects broader trends toward digital health and precision medicine [2]. Industry-driven tools—advanced monitoring, decision support, and predictive algorithms—are increasingly embedded in telemedicine infrastructures such as virtual ICUs (vICUs), enabling continuous remote monitoring of critically ill patients [3].

Observational and retrospective studies link vICUs to timely intervention, best practice adherence, and early recognition of deterioration [4–7]. Telemedicine has also been reported to broadly facilitate decision-making across diverse populations and settings [3,8], positioning it as a platform for AI integration amid rising patient complexity, staffing shortages, and escalating costs [9]. Yet major gaps remain. Implementing AI within Electronic Medical Record (EMR)-dependent environments poses challenges of interoperability, interface design, and workflow alignment [10,11]. Ethical concerns also persist regarding data privacy, transparency, and accountability, with inadequate disclosure risking clinician and patient mistrust [12,13].

Evidence linking AI or telemedicine directly to improved morbidity and mortality is limited. While some studies report gains in diagnostic accuracy or timeliness, robust data on mortality, ICU stay, or patient-centered outcomes are inconsistent. Pereira et al. found no reduction in ICU stay or mortality with tele-ICU deployment [14], whereas pre–post and stepped-wedge studies have reported improvements in process quality and, in some contexts, length of stay or mortality [15,16]. Conversely, a large cross-sectional analysis of mechanically ventilated patients found no association between hospital-level tele-critical care availability and improved outcomes [17]. Meta-analyses suggest benefits are more likely when tele-ICU teams have decision-making authority and in higher-risk settings [18,19], and recent state-of-the-art reviews highlight implementation context (authority, workflows, adoption) as key effect modifiers [20].

To address these gaps, we sought to systematically evaluate the impact of implementing an advanced AI-driven clinical decision support system in our academic medical setting. Specifically, we performed a before-and-after study to assess both immediate and sustained effects of AI integration. We hypothesized that integration of an EMR-connected vICU with predictive analytics would reduce ICU length of stay (LOS, primary outcome) and improve ventilation duration and vasopressor exposure (secondary outcomes) compared across pre-specified periods separated by a wash-out. Through this evaluation, we aimed to provide much-needed empirical evidence regarding the clinical value and operational feasibility of AI-driven tools in critical care, thereby informing best practices for future technological adoption.

## Methods

This study employed a before-and-after design to evaluate the clinical impact of a vICU platform (CLEW Medical Ltd, Netanya, Israel) on key clinical outcomes. Patient outcomes from the initial implementation period (October 2022–April 2023) were compared to those following the established program period (October 2023–April 2024). These windows were matched by calendar months to minimize seasonal variation in respiratory illness and ICU demand. We incorporated a 6-month washout after vICU go-live to allow adoption and workflow stabilization before outcomes assessment, consistent with prior tele-critical care and digital monitoring rollouts that use post-deployment washout windows (commonly ≈2 months [21,22]) prior to analysis. The analysis included patients admitted to three distinct units: a multispecialty surgical critical care unit (SAICU), a neurocritical care unit (NCCU), and an intermediate care (ICU step-down) unit.

Patients were eligible for inclusion if they required mechanical ventilation for at least 12 h, had an ICU LOS longer than 6 h, and a total hospital LOS of less than 30 days. De-identified patient data, with all health identifiers and dates removed, were provided by CLEW Medical Ltd. The primary outcome was ICU LOS. Secondary outcomes included hospital LOS, ICU readmission, ICU and in-hospital mortality, and the duration of mechanical ventilation and vasopressor therapy. APACHE II/SOFA were not uniformly available in the de-identified extract; we report case-mix index as a proxy and balance check (**Table 1**).

**Table 1:** General characteristics of critically ill patients included in the study.

	Initial Implementation period (n = 266)	Established program period (n = 264)	*P-*value
Age in years, mean ± SD	63.4 ± 18.2	61.2 ± 18.3	0.170
Male, n (%)	145 (54.5)	142 (53.4)	0.867
Weight in kilograms, mean ± SD	86.0 ± 27.6	88.8 ± 30.2	0.267
Case Mix Index, monthly avg (SE)	3.1 (0.5)	3.0 (0.5)	0.918

### Telehealth structure

Data from clinical monitors and from the Cerner Millenium electronic medical record (Oracle Health, Kansas City, MO) was structured and transmitted according to the Health Level Seven (HL7) standards. This included patient demographics, admission/discharge/transfer (ADT) events, laboratory test results, medication orders, radiology reports and billing and insurance information.

**Fig 1:**
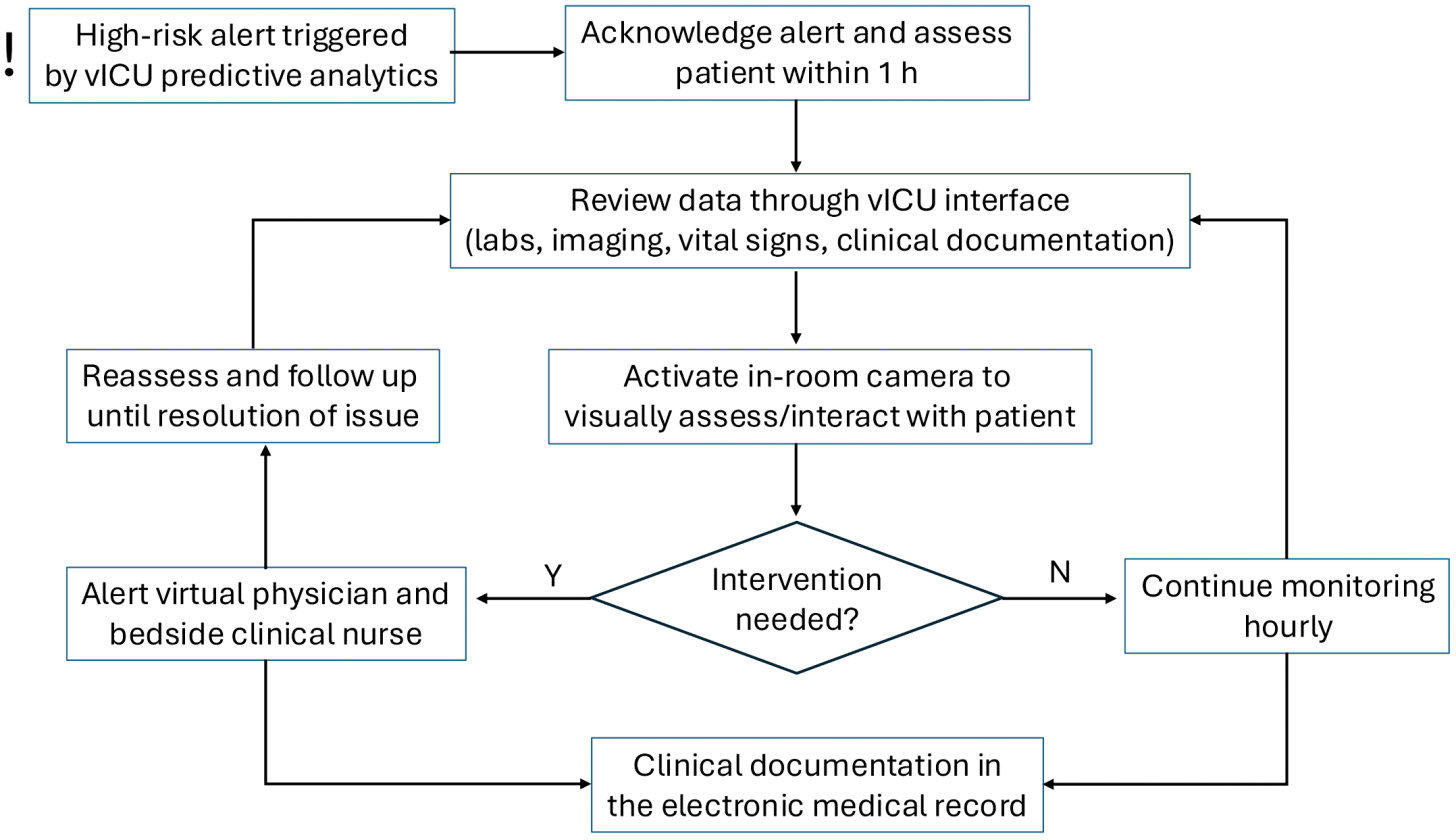
Schematic of the vICU workflow triggered by a “high-risk” alert for impending respiratory failure or hemodynamic instability.

The vICU platform ingests HL7 streams via a Rhapsody interface (Lyniate, Boston, MA) to a secure cloud service where proprietary time-series risk scores for impending respiratory failure or hemodynamic instability are computed using continuous and intermittent structured EMR data. Scores are rendered in a browser dashboard to the tele-critical care nursing team, who conduct routine safety rounds and escalate to bedside teams per unit protocol when thresholds or clinical patterns warrant (**Fig 1**). No facial recognition or image analytics were used. Investigators did not alter model parameters.

### Telehealth clinical care team

The vICU model is organized as a nurse-led tele-critical care program, with dedicated vICU physician oversight. The service provides continuous coverage across approximately 187 patient beds, spanning ICU, step-down units, and the emergency department. The team is composed of 23 critical care nurses with an average of 20 years of experience, over half of whom hold advanced certifications or leadership roles. These nurses provide audiovisual surveillance for newly admitted patients, perform routine safety rounds at regular intervals, and monitor high-risk patients using predictive analytic tools (**Fig 1**). The system is designed to screen large volumes of physiologic, laboratory, and clinical data, escalating concerns to the bedside team when appropriate. The structure emphasizes continuous monitoring and early identification of clinical deterioration, with the virtual nurses serving as an adjunct to bedside providers.

The range of services offered by the vICU nursing team includes admission intake support, hourly safety surveillance, and targeted monitoring for regulatory compliance metrics such as venous thromboembolism prophylaxis, anticoagulation therapy, and sepsis bundle adherence. Additional responsibilities include documentation during rapid response and code events, wound and skin assessments, and observation during selected bedside procedures.

The vICU also extends support to the emergency department, providing surveillance for critically ill patients awaiting bed placement. In 2024, this program’s vICU nurses documented nearly 6,000 hours of direct support to bedside staff, performed close to 500 admission assessments, and identified multiple patient safety concerns, including near-miss events that were corrected before harm occurred.

### Ethics approval and consent to participate

This study was exempt from ethical approval under Institutional Protocol HRP-310 (May 1, 2025), Human Studies Protection Office at Penn State College of Medicine. Because only de-identified clinical data were used, with no collection of Protected Health Information (PHI), no physical procedures or environmental manipulations, and no direct contact with participants, informed consent was not required.

### Data analysis

This pilot was designed for effect-size estimation rather than a definitive hypothesis test; thus, no a priori power calculation was performed. All eligible admissions in each window were included to estimate AUC/mean differences with 95% CIs and to inform sample-size planning for subsequent multicenter studies.

We compared the pre-implementation period with the established-implementation period using Welch’s t-tests for continuous outcomes and χ² tests for proportions. Because the epochs are calendar-matched (Oct–Apr in consecutive years), seasonality is minimized; therefore month fixed effects were not added. As a sensitivity analysis, we fit unit-adjusted models within the overall ICU cohort: ordinary least squares (HC3 robust SEs) for ICU and ventilation hours and logistic regression for vasopressor use ≥ 12 h, including a categorical unit term (SAICU, NCCU, multi, other). Adjusted contrasts are reported as post–pre differences with 95% CIs.

Because nurse-to-patient staffing ratios were not consistently available across units, we report monthly CMI (**Table 1**) as a case-mix proxy and acknowledge residual staffing as a study limitation. All hypothesis tests were two-sided, with a significance threshold of p < 0.05. All analyses were performed using R version 4.3.2 (R Foundation for Statistical Computing, Vienna, Austria).

## Results

**Fig 2:**
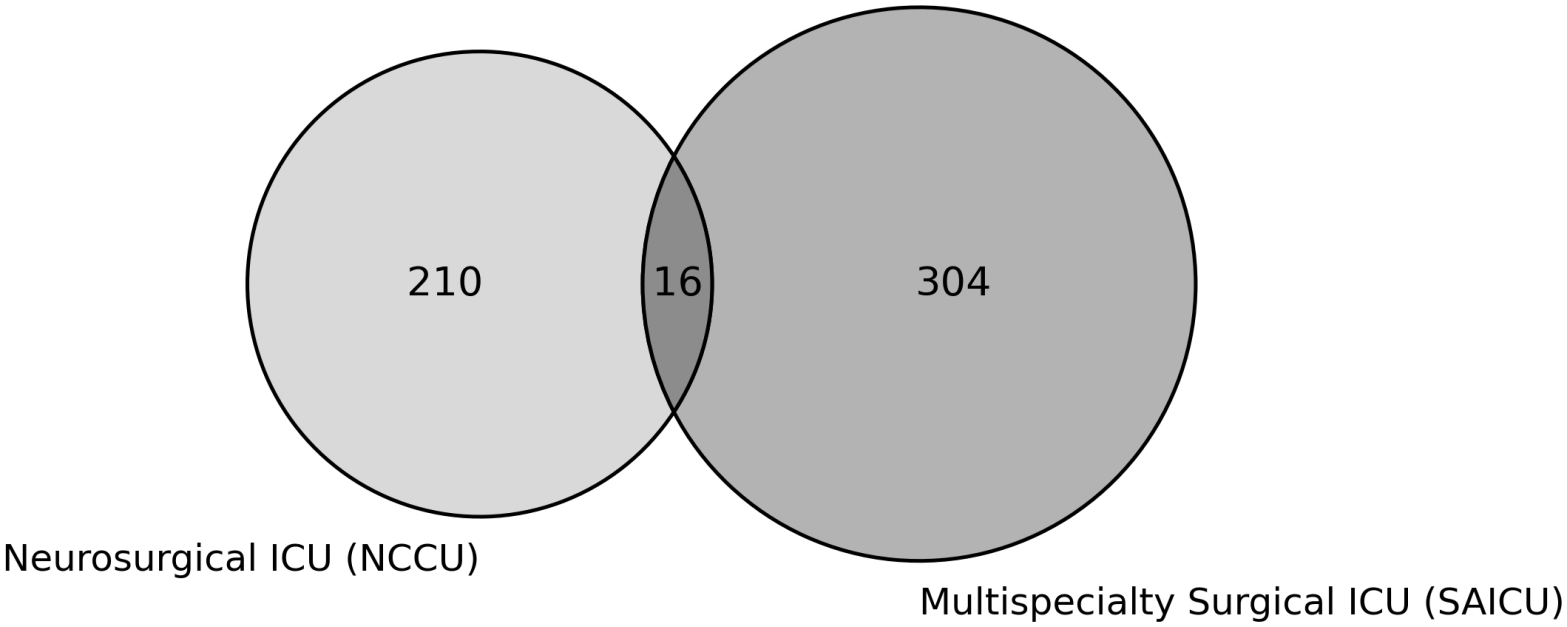
Patient distribution during the evaluation period, stratified by ICU cohort. The sum of the patients comprise the overall ICU cohort.

A total of 530 patients were included in the study, with 266 patients admitted during the initial implementation period and 264 during the established program period (**Fig 2**). Baseline characteristics, including age, gender, weight, and case-mix index, were comparable between periods (**Table 1**).

**Fig 3:**
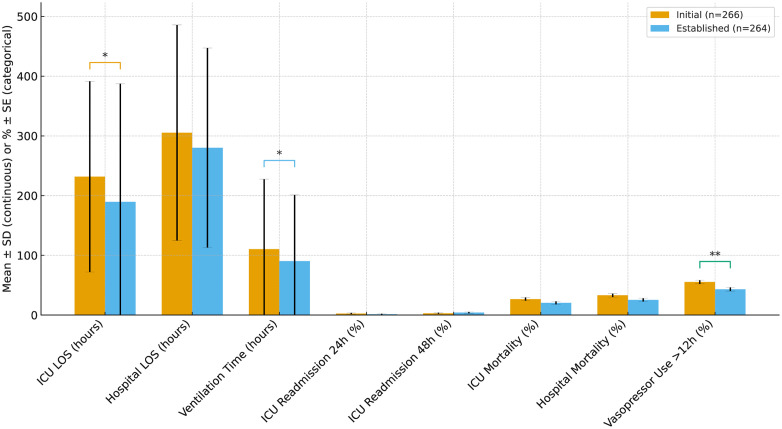
ICU outcomes before vs. after implementation of a virtual ICU (vICU) platform. Bar charts display mean ± standard deviation (SD) for continuous outcomes (ICU length of stay, hospital length of stay, ventilation time) and percentage ± binomial standard error (SE) for categorical outcomes (ICU readmissions, mortality, vasopressor use). Light blue bars represent the initial implementation period (October 2022–April 2023; *n* = 266), and dark blue bars represent the established program period (October 2023–April 2024; *n* = 264). Significance brackets indicate results of statistical testing (Welch’s *t*-test for continuous variables, χ² test for proportions). *p* < 0.05 (*), *p* < 0.01 (**).

In the overall ICU cohort (HMC ICUs; n = 530; pre = 266, post = 264), mean ICU LOS decreased from 232 h to 198 h (Δ −34 h; p = 0.011, **Fig 3**). Mechanical ventilation duration decreased from 110 h to 90 h (Δ −20 h; p = 0.044). The proportion receiving vasopressors ≥12 h declined from 55% to 43% (Δ −12 percentage points; p = 0.007). Hospital LOS decreased numerically (305 h to 280 h; Δ −25 h) but was not statistically significant (p = 0.097). In-hospital mortality (33% → 25%; p = 0.064), ICU mortality (27% → 21%; p = 0.112), and 24/48-h readmissions did not differ significantly. These significant changes in ICU and ventilation hours are consistent with earlier physiologic stabilization.

In unit-adjusted models within the overall ICU cohort (controlling for SAICU/NCCU), the post-implementation period remained associated with shorter ICU LOS (adjusted Δ −33 h, 95% CI −59 to −7; p = 0.01), shorter ventilation duration (adjusted Δ −21 h, 95% CI −40 to −2; p = 0.03), and lower vasopressor use ≥ 12 h (adjusted Δ −12 pp, 95% CI −21 to −3. p = 0.01). Effects were largest in surgical-dominant units, with expected attenuation when pooling across units.

**Fig 4:**
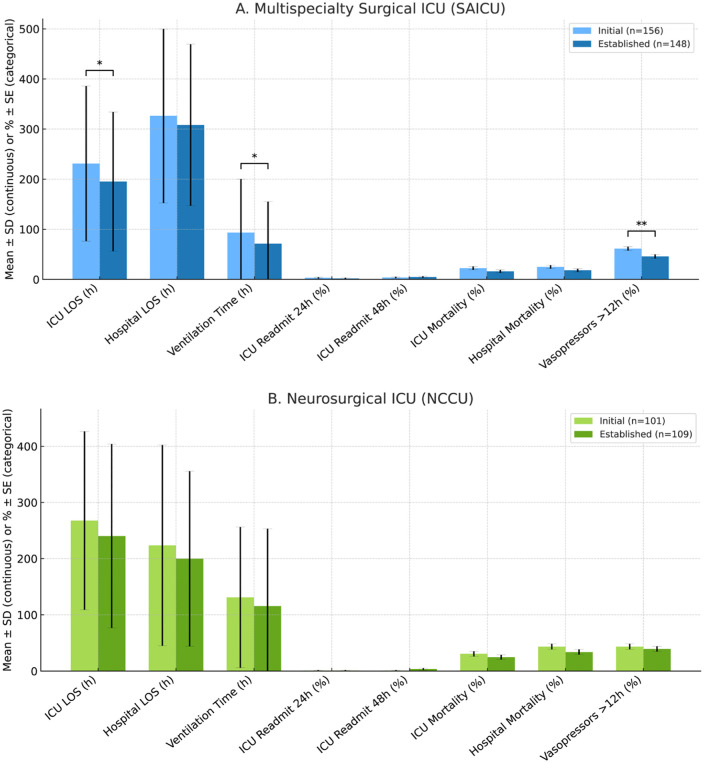
Outcomes before vs after implementation of a virtual ICU (vICU) program in two units. **(A)** Multispecialty Surgical ICU (SAICU). **(B)** Neurosurgical ICU (NCCU). Bars show mean ± SD for continuous outcomes (ICU length of stay, hospital length of stay, and ventilation time) and percentage ± binomial standard error (SE) for categorical outcomes (ICU readmission at 24 h and 48 h, ICU mortality, hospital mortality, and vasopressor use > 12 h). Light bars denote the initial implementation period (SAICU *n* = 156; NCCU *n* = 101) and dark bars denote the established program period (SAICU *n* = 148; NCCU *n* = 109). Significance brackets indicate hypothesis tests comparing periods (Welch’s *t*-*t*est for continuous variables; χ² test for proportions). Significance thresholds: *p* < 0.05 (*), p < 0.01 (**), p < 0.001 (***).

Analysis of the multispecialty surgical ICU (SAICU) subgroup also demonstrated significant reductions in mean ICU LOS (231–195 h, p = 0.034), ventilation time (93–72 h, p = 0.047), and vasopressor use for more than 12 h (62% to 46%, p = 0.009). Hospital LOS, ICU and hospital mortality, and readmission rates remained statistically unchanged (**Fig 4A**).

In the NCCU subgroup, no statistically significant differences in ICU or hospital LOS, mortality rate, duration of mechanical ventilation, vasopressor use, or ICU readmissions were observed between periods (**Fig 4B**). Similarly, no significant differences were noted in hospital LOS, mortality, or ICU admissions within 24 and 48 h in patients cared for in the step-down unit between periods (Table 2).

**Table 2:** Length of stay, mortality, readmission rates, ventilation, and vasopressor outcomes for the Intermediate Level of Care (ICU step-down) Unit.

Outcome	Initial Implementation period (n = 87)	Established program period (n = 109)	*P-*value
Hospital LOS, mean no. of hours ± SD	240.2 ± 167.2	229.0 ± 162.3	0.636
ICU admission within subsequent 24 h, n (%)	16 (18.4%)	16 (14.7%)	0.614
ICU admission within subsequent 48 h, n (%)	16 (18.4%)	16 (14.7%)	0.614
Hospital mortality, n (%)	32 (36.8%)	39 (35.8%)	1.0

## Discussion

This study demonstrates that implementation of a cloud-based vICU program was associated with improvements in clinical outcomes among acutely ill patients. Specifically, we observed reductions in ICU LOS, duration of mechanical ventilation, and vasopressor use following vICU deployment. These improvements were also seen in the SAICU subgroup, suggesting broad responsiveness to virtual oversight and support. They may have resulted from prompt recognition of patient decline, better care coordination, and earlier withdrawal of intensive therapies which were facilitated by remote monitoring and AI-driven tools. Importantly, the results were achieved without significant changes in hospital LOS, ICU or hospital mortality, or ICU readmission rates. This suggests that the vICU program may selectively enhance the management of high-acuity events and expedite clinical stabilization without negatively impacting broader patient outcomes. Specifically, the absence of mortality difference with reduced ICU support duration is consistent with safety while improving efficiency. Findings were robust to case-mix differences across ICU types; adjusting for unit preserved the magnitude and significance of ICU LOS, ventilation, and vasopressor effects. Because this was a single-center before-and-after evaluation using de-identified operational data, the findings should be interpreted primarily as evidence of feasibility, safety, and potential efficiency gains rather than definitive proof of causal benefit.

While this study did not include a formal economic evaluation, the observed reductions in ICU and ventilation time—together with vICU activity (≈6,000 hours of direct tele-critical care support and ≈500 admission assessments)—are consistent with potential capacity gains (greater bed turnover, shorter time on invasive support) and workload redistribution (centralized surveillance, earlier escalation, targeted bedside tasks). In nurse-constrained settings, continuous virtual monitoring can help prioritize bedside attention during peak demand, potentially reducing unplanned escalations, after-hours overtime, and sitter utilization. To quantify these effects, we plan a prospective cost-effectiveness analysis from the hospital perspective, using micro-costing of vICU staffing/infrastructure against changes in ICU/ventilator days, transfer delays, and complication rates (e.g., ventilator-associated pneumonia, CLABSI, pressure injury), with sensitivity analyses across census, staffing mix, and adoption levels. A parallel budget-impact analysis over 12 months will estimate net costs or savings, and we will link process measures (alerts per patient-day, time-to-acknowledgment, time-to-intervention) to outcomes to identify the pathways by which value is realized.

Our findings align with mounting evidence that telemedicine ICU programs improve critical care efficiency and patient outcomes by enhancing monitoring and accelerating decision-making. For instance, McCambridge et al. reported a significant reduction in mechanical ventilation use from 36% to 32% following the implementation of a tele-ICU system [23]. Tele-ICU–led ventilator rounds have also been shown to improve adherence to lung-protective ventilation strategies, resulting in significant reductions in ventilator duration and ICU LOS [24]. Similarly, tele-ICU implementation can shorten ICU length of stay and ventilation duration, while also maintaining workflows and reducing EMR-related tasks for bedside clinicians [25]. They improve compliance with best practice protocols along with preventive measures to help lower complications and standardize care [7,24].

Although multiple studies [7,23–25] have reported reductions in ICU mortality following tele-ICU implementation, we did not observe a similar association in our cohort. Several factors may explain this discrepancy. Differences in patient populations could be influential; our cohort may have had a more heterogeneous case mix, which could dilute the observable effect. Additionally, variation in ICU organizational structures—such as the degree of integration between vICU teams and bedside staff, staffing ratios, and institutional protocols—may influence the effectiveness of remote interventions.

Absolute gains were largest in surgical-dominant settings, suggesting that context-specific integration may amplify benefits. Subgroup analysis suggested that observed benefits were largely driven by our multispecialty surgical ICU, whereas the NCCU and step-down unit showed no statistically significant changes in primary or secondary outcomes. We hypothesize three complementary reasons. First, actionability: surgical ICU trajectories (post-operative bleeding, ileus, fluid shifts, early respiratory failure) generate frequent, protocol-addressable alerts with short decision latencies; standardized pathways (e.g., early mobilization, ventilator weaning, analgesia/sedation titration) may have amplified the impact of continuous remote surveillance. Second, signal alignment: the monitored variables feeding the risk scores (vitals, invasive pressures, ventilator parameters, labs) map closely onto surgical deterioration modes but may be less sensitive to neurologic trajectory in the NCCU, where bedside neurologic examination and imaging drive many decisions. This difference in signal relevance likely reduced alert specificity and limited opportunities for high-yield escalations in the NCCU. Third, workflow context: SAICU teams may have had higher adoption of virtual escalation scripts and faster order execution (e.g., fluid challenges, vasoactive adjustments, ventilator changes), while the NCCU’s individualized targets and the step-down unit’s lower baseline acuity reduced opportunities for remote oversight to change outcomes. Thus, variation in workflow adoption likely contributed to the differential impact across units.

These unit-level contrasts suggest that the effectiveness of tele-ICU integration depends on the match between alert content and unit-specific action pathways, and they motivate targeted optimization (unit-tailored thresholds, neurologic data elements, and escalation scripts) rather than a one-size-fits-all deployment. Prospective work should include heterogeneity-of-treatment-effect analyses with unit-intervention interaction terms and process measures (alerts per patient-day, time-to-acknowledgment, time-to-intervention, adherence to escalation scripts) to determine whether unit-specific adoption and alert actionability mediate outcome differences.

This study has several strengths that support the reliability of its findings. The before-and-after design included clearly defined pre- and post-implementation periods, along with a wash-out phase to reduce carry-over effects. Because the two study epochs covered identical calendar months in consecutive years, residual seasonality is unlikely to explain the results. Consistent inclusion criteria and a standardized analytical approach ensured balanced comparisons between cohorts. Inclusion of multiple hospital ward types provided a broader view of the intervention’s impact across varied clinical environments. However, several limitations should be considered. The single-center design may limit generalizability. Detailed nurse-to-patient staffing ratios were unavailable across units, precluding direct adjustment for staffing patterns. We partially mitigated confounding through a unit-adjusted model; however, residual confounding by staffing and workflow remains possible. Additionally, the study did not capture detailed data on the frequency, timing, or nature of virtual clinician involvement. A Hawthorne effect—practice changes due to heightened observation during implementation—may partly explain early improvements despite our wash-out period. These limitations emphasize the importance of future studies that examine process measures to clarify the impact of vICU programs on outcomes.

## Conclusions

In this single-center evaluation, an AI-enabled vICU program appeared feasible and safe, and was associated with reductions in ICU stay, ventilator duration, and vasopressor exposure. These findings suggest potential efficiency gains, though causal inference is limited by the study design. Operationally, vICUs extend intensivist coverage, reduce care delays, and may ease nursing workload, though success depends on local workflow alignment and adequate training. Future prospective studies and randomized trials are needed to isolate the effects of virtual engagement, AI-driven risk stratification, and remote decision-making. Beyond mortality or LOS, evaluations should include patient-centered outcomes (e.g., functional recovery, quality of life), clinician/patient satisfaction, and cost-effectiveness to determine whether vICUs are both clinically effective and operationally sustainable across diverse healthcare environments.

## Supporting information

S1 Data Raw Outcomes Data by period and clinical care unit. (ZIP)
